# Classification Models for Neurocognitive Impairment in HIV Infection Based on Demographic and Clinical Variables

**DOI:** 10.1371/journal.pone.0107625

**Published:** 2014-09-19

**Authors:** Jose A. Muñoz-Moreno, Núria Pérez-Álvarez, Amalia Muñoz-Murillo, Anna Prats, Maite Garolera, M. Àngels Jurado, Carmina R. Fumaz, Eugènia Negredo, Maria J. Ferrer, Bonaventura Clotet

**Affiliations:** 1 Fundació Lluita contra la SIDA - Hospital Universitari Germans Trias i Pujol, Badalona, Catalonia, Spain; 2 Universitat Autònoma de Barcelona, Cerdanyola del Vallès, Catalonia, Spain; 3 Universitat Politècnica de Catalunya, Barcelona, Catalonia, Spain; 4 Universitat de Barcelona, Barcelona, Catalonia, Spain; 5 Consorci Sanitari Hospital de Terrassa, Terrassa, Catalonia, Spain; 6 Grup de Recerca Consolidat de Neuropsicologia - Universitat de Barcelona, Barcelona, Catalonia, Spain; 7 Institut de Recerca per la SIDA IrsiCaixa, Badalona, Catalonia, Spain; 8 Universitat de Vic, Vic, Catalonia, Spain; University of Athens, Medical School, Greece

## Abstract

**Objective:**

We used demographic and clinical data to design practical classification models for prediction of neurocognitive impairment (NCI) in people with HIV infection.

**Methods:**

The study population comprised 331 HIV-infected patients with available demographic, clinical, and neurocognitive data collected using a comprehensive battery of neuropsychological tests. Classification and regression trees (CART) were developed to obtain detailed and reliable models to predict NCI. Following a practical clinical approach, NCI was considered the main variable for study outcomes, and analyses were performed separately in treatment-naïve and treatment-experienced patients.

**Results:**

The study sample comprised 52 treatment-naïve and 279 experienced patients. In the first group, the variables identified as better predictors of NCI were CD4 cell count and age (correct classification [CC]: 79.6%, 3 final nodes). In treatment-experienced patients, the variables most closely related to NCI were years of education, nadir CD4 cell count, central nervous system penetration-effectiveness score, age, employment status, and confounding comorbidities (CC: 82.1%, 7 final nodes). In patients with an undetectable viral load and no comorbidities, we obtained a fairly accurate model in which the main variables were nadir CD4 cell count, current CD4 cell count, time on current treatment, and past highest viral load (CC: 88%, 6 final nodes).

**Conclusion:**

Practical classification models to predict NCI in HIV infection can be obtained using demographic and clinical variables. An approach based on CART analyses may facilitate screening for HIV-associated neurocognitive disorders and complement clinical information about risk and protective factors for NCI in HIV-infected patients.

## Introduction

Neurocognitive impairment (NCI) is a concern in HIV infection. Between one-third and two-thirds of HIV-infected individuals experience this complication [1], which may lead to impaired daily functioning [2], poor quality of life [3], difficulties in clinical management (eg, poorer adherence to antiretroviral therapy) [4], and even higher death rates [5]. Rapid, reliable, and feasible detection of NCI is therefore a key element in the therapeutic management of HIV-infected patients.

Neuropsychological test batteries are widely recommended for assessment of neurocognitive status in HIV-infected persons [6]–[9]. However, they have been proposed mainly for comprehensive evaluation of neurocognitive functioning, rather than rapid detection of impairment in clinical practice. Definitive diagnosis of HIV-associated neurocognitive disorders (HAND) is important, and new approaches are necessary to facilitate screening [10]. Access to neuropsychological resources varies depending on the availability of care services for HIV-infected people, and practical screening tools are not regularly used for detection of HIV-related NCI. In this regard, additional methods may help to assist in the identification of these disorders in HIV-infected individuals.

Demographic and clinical data are easily collected and could help to identify NCI during the routine care of HIV-infected patients, since they provide crucial information not only about physical status, but also about central nervous system (CNS) functioning [7], [9]. Therefore, we recorded demographic and clinical variables in order to design accurate and practical models for predicting NCI in patients with HIV infection in daily clinical practice. We used the classification and regression trees (CART) methodology, which allowed us to obtain visual and easy-to-read trees with variable accuracy for predicting the existence of NCI.

## Methods

### Design and Study Population

The study was based on data from 331 HIV-infected outpatients attended at 7 HIV units in Barcelona, Catalonia, Spain. Individuals were selected because they had voluntarily undergone a comprehensive neuropsychological test battery, which was offered routinely in the daily clinical practice of the participating centres. Only participants with available demographic and clinical data were included. The remaining inclusion criteria were age ≥18 years and positive results for HIV infection in ELISA and Western-blot. Participants whose data were incomplete for any of the study variables were excluded. All participants provided their written informed consent to undergo neurocognitive assessment, and the Ethics Committee of Germans Trias i Pujol University Hospital (Badalona, Barcelona, Catalonia, Spain) approved the study. Data were collected from September 2009 to March 2013.

### Demographic and Clinical Assessments

Demographic variables included age, gender, route of transmission, employment status, and years of education. Data were self-reported.

The clinical variables collected were current CD4 cell count, nadir CD4 cell count, plasma viral load, past highest viral load, previous or no experience of antiretroviral therapy, current antiretroviral therapy, CNS penetration-effectiveness (CPE) score, previous treatment interruptions, time since HIV diagnosis, time since initiation of the first regimen, time on the current regimen, coinfection with hepatitis C virus (HCV), AIDS condition, and existence of comorbidities with the potential to affect neurocognitive performance. Data were retrieved from medical reports and local clinical databases at the study sites. The CPE score was calculated using the proposal by Letendre and collaborators [11]. Treatment interruption was defined as discontinuation of antiretroviral therapy for more than 15 days, regardless of the number of discontinuations, and was recorded based on results from our group revealing connections between previous interruptions and worse neurocognitive status [12]. Potential confounding comorbidities were established mainly according to criteria of the Frascati Group [7] and were treated as a categorical variable (yes/no). The comorbidities included lifetime or current diagnosis of a psychiatric disorder, receiving psychopharmacologic therapy, drug or alcohol abuse, and prior or current CNS-related disease. Emotional status, that is, specifically symptoms of depression and anxiety, was assessed using the Beck Depression Inventory (BDI) [13] and the State-Trait Anxiety Inventory (STAI) [14]. The cognitive-affective subscale of the BDI was used to avoid biases related to somatic symptoms [15].

### Neurocognitive Assessment

Neurocognitive functioning was evaluated using a neuropsychological battery composed of 14 tests covering 7 domains that are recommended for assessment in HIV infection [7], [9], as follows: the Letter-Numbers and Digits tests of the Wechsler Adult Intelligence Scale-III (WAIS-III) [16] for attention/working memory; part A of the Trail Making Test (TMT) [17] and the Symbol Digit Modalities Test (SDMT) [18] for information processing speed; the California Verbal Learning Test - Part II (CVLT) [19] for verbal memory and learning; part B of the TMT [17], the Stroop Test [20], the Wisconsin Card Sorting Test (WCST) [21], and the Tower of London (TOL) test [22] for executive function; the Controlled Oral Word Association Test (COWAT) [23] and the Animals test [24] for verbal fluency; and the Grooved Pegboard Test (GPT) [25] for motor function. In addition, the Vocabulary test of the WAIS-III [16] was used to assess premorbid intelligence. Self-reported cognitive complaints were also recorded and considered as a dichotomous categorical variable (yes/no) in cases where patient reported symptoms of impairment in memory, attention, or planning [26].

NCI was determined based on a neuropsychological criterion, which was defined as performing at least 1 standard deviation below the normative mean in at least 2 neurocognitive areas, as extensively reported elsewhere [1], [7], [9]. Standardized T scores were then used for all the analyses. These were obtained by standardizing raw scores according to available normative data, mainly covering age, gender, and years of education.

The classification of HAND was also used to describe the study sample and was obtained by applying the Frascati criteria [7], using interference in daily functioning in terms of self-reported assessment, which was expressed as a dichotomous categorical variable (yes/no).

### Data Analyses

We used a tree-structured approach to provide useful classification models for predicting NCI in HIV-infected individuals from a clinical practice perspective. Specifically, we used CART analyses, a methodology that generates rules for decisions involving categorical outcomes [27].

CART analyses are based on the successive selection of a variable and its value to subdivide the individuals comprising a sample so as to better classify them. Consequently, the criteria for subdivision are variables that make it possible to reduce the difference in scores. This approach has several advantages. First, CART analysis generates structured and visual classification rules, which facilitate understanding for the study findings. Second, the resulting outcomes depend on the different levels of accuracy. Third, as for quantitative variables, CART provides specific cut-off points that inform the user about key values that can affect the final outcome. Fourth, as a non-parametric method, CART analyses are not affected by the data distribution and thus enable rigorous assumptions to be established without the undesirable effects produced by outliers, collinearity, or heteroscedasticity. Therefore these characteristics make it possible to apply classification variables with different origins and a specific typology (e.g., categorical or continuous variables).

In our study, the presence of NCI was the main study variable and was therefore considered a final endpoint in the classification models, namely, presence or absence of a disorder. The cross-validation method was used to determine the optimal size of the tree as a function of misclassification and of the cost-complexity parameter, depending on which the initial tree was pruned to obtain a simpler classification rule.

Because we followed a practical clinical approach, models were elaborated separately for antiretroviral treatment-naïve and treatment-experienced patients, and we finally selected those with the greatest accuracy and simplicity according to the accuracy of classification found and the number of terminal nodes displayed.

All study analyses were performed using SPSS for Windows, version 15.0 (Chicago, SPSS Inc.) and R, version 2.11 (http://www.r-project.org).

## Results

### Treatment-Naïve Patients

Of the total sample of 331 patients, 52 were treatment-naïve. Patients in this group were predominantly men (85%), middle-aged (median [interquartile range]: 35 years [28]–[42]), with a median viral load of 17,000 copies/mL (4,950–61,250), current CD4 count of 437 cells/µL (292–693), and nadir CD4 count of 416 cells/µL (232–597). NCI was present in 21 of the 52 treatment-naïve individuals (40%), and cognitive complaints were reported in 11 of the 21 (52%). The remaining demographic, clinical, and neurocognitive characteristics are shown in ***Table 1***.

**Table 1:** Characteristics of the study sample.

		Treatment-naïve patients (n = 52)	Treatment-experienced patients (n = 279)
**Age, years**		35 (28–42)	44 (40–50)
**Male,%**		85	83
**Infection route,%**			
	**Injecting drug user**	4	21
	**Heterosexual**	15	19
	**Men who have sex with men**	65	48
	**Other**	4	1
	**Unknown**	12	11
**Education, years**		12 (10–15)	12 (8–15)
**Time since HIV diagnosis, years**		1 (0–3)	11 (6–17)
**On ART,%**		-	100
**Time on treatment, years**		-	8 (3–12)
**Time on current regimen, months**		-	12 (0–24)
**Previous ART interruptions,%** a		-	36
**CD4 cell count, cells/µL**		437 (292–693)	539 (383–737)
**Nadir CD4 cell count, cells/µL**		416 (232–597)	189 (87–313)
**Plasma viral load, copies/mL**		17,000 (4,950–61,250)	50 (50–50)
**Undetectable viral load,%** b		5	86
**Past highest viral load, copies/mL**		41,000 (5,861–132,500)	92,500 (17,000–240,000)
**Coinfection with HCV,%**		9	24
**Standardized depression score** c		52 (42–58)	48 (37–57)
**Standardized anxiety score** d		52 (41–59)	48 (40–57)
**Standardized premorbid intelligence score** e		53 (43–60)	56 (50–60)
**Neurocognitive impairment,%**		40	50
**HAND,%** f			
	**ANI**	50	53
	**MND**	50	46
	**HAD**	0	1
**Cognitive complaints,%** f		52	53
**Potential confounding comorbidities,%** g		32	39

Data expressed as median (interquartile range), except when indicated otherwise.

Standardized scores are adjusted for age, gender and education, according to available normative data.

Abbreviations: ANI, asymptomatic neurocognitive impairment; ART, antiretroviral therapy; HAD, HIV-associated dementia; HAND, HIV-associated neurocognitive disorder; HCV, hepatitis C virus; MND, mild neurocognitive disorder.

*^a^*ART interruption defined as discontinuing therapy>15 days at any time for any reason in the past.

*^b^*Undetectable at ≤40 copies/mL level.

*^c^*Depression scores based on the Beck Depression Inventory test.

*^d^*Anxiety scores based on the State-Trait Anxiety Inventory.

*^e^*Premorbid intelligence scores based on the Vocabulary test from the Wechsler Adult Intelligence Scale-III.

*^f^*From patients with neurocognitive impairment.

*^g^*Potential confounding comorbidities defined as lifetime or current diagnosis of a psychiatric disorder, receiving psychopharmacologic therapy, drug or alcohol abuse, and prior or current CNS-related disease.

**Figure 1:**
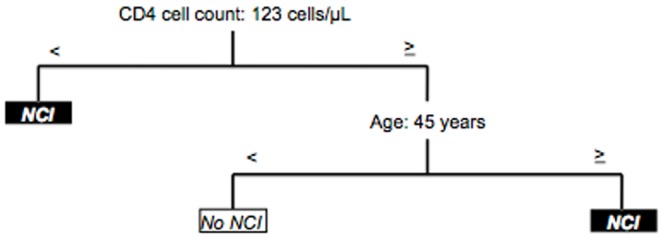
Classification model for treatment-naïve patients (n = 52). Correct classification: 79.6%, 3 final nodes.

The highest correct classification obtained from the trees was 83.6% in a model with 7 final nodes. This included 5 main variables: CD4 cell count, age, employment status, nadir CD4 cell count, and current CD4 cell count (by order of relevance). The simplest tree had 3 final nodes and included only CD4 cell count and age as predictive variables (***Figure 1***). The percentage of correct classification obtained from this tree was 79.6%. As for specific cut-off points, NCI was detected when the CD4 cell count was <123 cells/µL. When the CD4 cell count was ≥123 cells/µL, presence of of NCI varied with age: age ≥45 years was associated with impairment, and age <45 years was associated with non-altered functioning.

### Treatment-Experienced Patients

A total of 279 patients had previously received antiretroviral therapy and were all currently on treatment. Most were men (83%) and middle-aged (median, 44 years [40–50]), with an undetectable viral load (86%), a median current CD4 count of 539 cells/µL (383–737), and a nadir CD4 count of 189 cells/µL (87–313). NCI was present in 140 individuals (50%), of whom 71 (53%) reported complaints. The remaining characteristics are displayed in ***Table 1***.

The highest correct classification in the trees obtained was 83.5% in a model with 12 final nodes, including a total of 8 variables: years of education, CPE score, nadir CD4 cell count, age, employment status, years since HIV diagnosis, years on antiretroviral therapy, and presence of potential confounding comorbidities.

**Figure 2:**
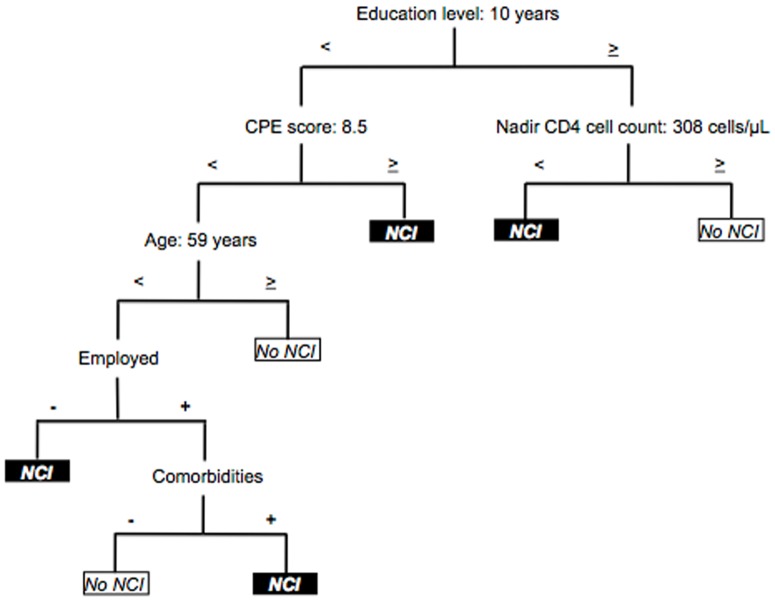
Classification model for treatment-experienced patients (n = 279). Correct classification: 82.1%, 7 final nodes.

Pruning the initial model provided a simple tree that did not substantially increase the misclassification rate. In a model with 7 final nodes and 6 variables, a correct classification of 82.1% was reached. The variables included in this tree were education, CPE score, nadir CD4 cell count, age, employment status, and existence of comorbidities (***Figure 2***). Years of education was the most relevant parameter. When this value was ≥10 years, the nadir CD4 cell count was the variable that established the presence of NCI: counts of ≥308 cells/µL were associated with absence of dysfunction and lower values with normal performance. However, when duration of education was <10 years, CPE score was found at the second level of prediction. In this regard, values of ≥8.5 were associated with impaired functioning. At a lower CPE score, age was revealed as a next parameter. An age ≥59 years was associated with normal performance; in younger patients, employment status was the variable that established the presence of NCI. Unemployment was associated with impairment; employment was associated with the existence of comorbidities at the lowest level of this model. Impairment was found in the presence of comorbidities and normal functioning in the absence of comorbidities.

**Figure 3:**
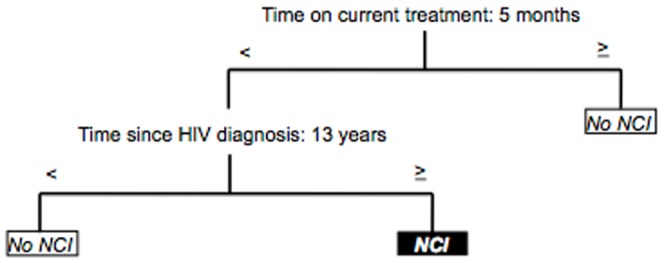
Classification model for treatment-experienced patients with undetectable viral load (n = 240). Correct classification: 73.4%, 3 final nodes.

In our search for a homogeneous subset of patients, we also studied individuals with an undetectable viral load (n = 240). The simplest model from the trees comprised 3 nodes and 2 variables and had a 73.4% correct classification. Months on current antiretroviral treatment and years since HIV diagnosis were the variables included. NCI was not predicted when patients had been on current therapy for ≥5 months. In patients with <5 months on treatment, the number of years since HIV diagnosis determined the presence of NCI. Impairment was found in people diagnosed with HIV infection ≥13 years age, but not in those diagnosed <13 years ago (***Figure 3***).

**Figure 4:**
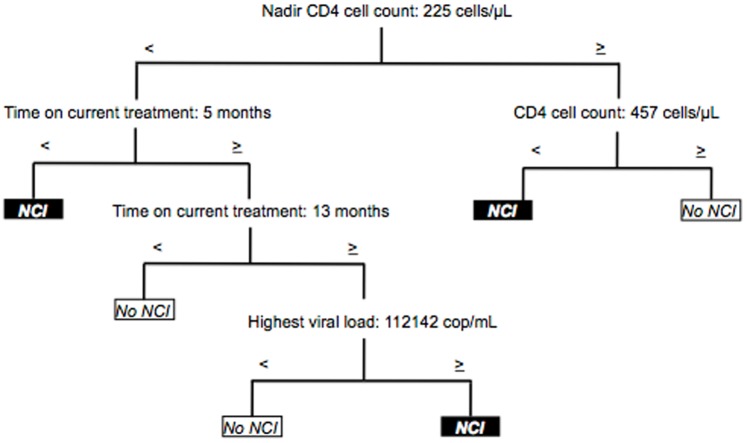
Classification model for treatment-experienced patients with undetectable viral load and no confounding comorbidities (n = 148). Correct classification: 88%, 6 final nodes.

Analysis of an even more homogenous subgroup—individuals with an undetectable viral load and no presence of potential confounding comorbidities (n = 148)—revealed remarkable accuracy with a correct classification of 88%, included 6 final nodes, and comprised 4 main predicting variables: nadir CD4 cell count, current CD4 cell count, months on current treatment, and past highest viral load (***Figure 4***). Nadir CD4 cell count was the first predictive variable. When this was ≥225 cells/µL, the current CD4 cell count was the second explanatory variable: normal functioning was observed at values of ≥457 cells/µL, and impairment was observed at lower values. With respect to nadir counts <225 cells/µL, time on current treatment and past highest viral load were associated with the presence of NCI. When time on treatment was <5 months, NCI was detected. In contrast, when time on treatment was between ≥5 and <13 months, impairment was not detected. And finally, when time on therapy was ≥13 months, past highest viral load value was associated with dysfunction. As for viral load, values ≥112,142 copies/mL were associated with NCI; lower values indicated normal performance.

## Discussion

We used a practical approach to analyze demographic and clinical data that could predict NCI in patients with HIV infection. Our data complement current knowledge on HIV-associated NCI, a major concern in the therapeutic management of HIV-infected patients. Different classification models were obtained, and we provide those that are potentially more useful in clinical practice, specifically in terms of accuracy and simplicity. Accuracy was based on the level of correct classification, and simplicity on the number of variables included in the models and the number of final nodes.

In our sample of antiretroviral-naïve patients, the variables most significantly identified as predictors of impairment were CD4 cell count and age. CD4 cell count is widely acknowledged as an optimal marker of progression of HIV infection and our observations confirm this fact. As our approach was clinical, we applied graphical statistical methodology, which provided clear and specific visual study outcomes. Accordingly, classification trees obtained for naïve patients revealed cut-offs of 123 cells/µL and age 45 years. The value for the CD4 cut-off is consistent with previous findings indicating comparable links between worse immunological status and existence of neurocognitive disruption, particularly in advanced immunosuppression [28], [29]. Similarly, older age is considered a risk factor for major cognitive changes in HIV-infected individuals, although a representative cut-off point has not been established in this regard [30], [31]. Nevertheless, most thresholds investigated have consisted of a cut-off at around age 50 years, which is fairly close to our results.

In patients on antiretroviral therapy, relevant parameters were years of education, nadir CD4 cell count, and CPE score. Education appeared at the first level of prediction. In fact, even in more complex models (>10 final nodes, not shown in the present report), level of education proved consistently relevant for determination of NCI. Greater education has been shown to act as a protective factor in the development of neurocognitive complications [32]. Irrespective of HIV infection, this factor has been considered a key variable in CNS-related diseases such as brain injury [33], Alzheimer's disease [34], and Parkinson's disease [35]. Similarly, nadir CD4 cell count is a key predictor of NCI in HIV infection [36]–[38]. Our observations confirm this association, suggesting a significant cut-off of <308 cells/µL for onset of NCI. In contrast, for the CPE score, previous findings do not appear to support ours [39]–[43]. As described above, our results suggest that higher CPE scores may be associated with a higher probability of impairment. In this sense, our hypotheses have been put forward in a clinical setting that is more difficult to manage, where more antiretrovirals are prescribed and more antiretroviral-induced neurotoxicity may exist. Nonetheless, our objectives and study design have not enabled us to draw solid findings. Conclusions on the CPE score should be obtained from prospective studies specifically designed to investigate this topic, after controlling for variables related to antiretroviral therapy and the characteristics of the population studied [9]. A similar effect occurs with age, since our results indicate that it could protect against NCI. Successful aging and a more robust cognitive reserve could act as protective factors, as reported elsewhere [44], [45]; however, again, we are unable to draw further conclusions. Our findings also show the importance of employment status and potential confounding comorbidities as determinants of neurocognitive dysfunction. Both features are consistent with previous observations on connections between active employment status and greater protection of CNS functioning [46], and between presence of potential comorbidities for NCI and impaired cognitive functioning [7].

We performed separate analyses of patients with undetectable viral load and patients with an undetectable viral load and no confounding comorbidities in order to investigate further the mechanisms of HIV-associated NCI. Significant differences in time variables were found in patients with an undetectable viral load: time on current antiretroviral treatment and time since HIV diagnosis were the main predictors. Both variables have previously been shown to play a key role in HIV-related cognitive decline, with theoretical advantages for longer time on therapy and disadvantages for a more extended time since HIV diagnosis [11], [47]. In the case of individuals with an undetectable viral load and no confounding comorbidities—apart from time on current treatment and nadir CD4 cell count—current CD4 cell count and past highest viral load were also key variables. Thus, according to CART, we confirmed the role of factors traditionally linked to cognitive decline in HIV infection.

Our study has several limitations. First, although we aimed to obtain a large sample, our sample size was reduced by performing separate analyses of 2 groups (treatment-naïve and treatment-experienced patients) and because of the number of subjects studied for each subgroup in the final nodes. As mentioned above, the statistical methodology applied was based on CART; consequently, the sizes for the category groups were further reduced. A second limitation is the clinical variables analyzed. Because we used available medical data from the participating centres, valuable supplementary clinical information (even if not systematically gathered) was ignored for the purposes of the study. Examples include cardiovascular risk factors and hormonal markers, which are known to be associated with cognitive dysfunction in both the general population and HIV-infected individuals. Another limitation is the use of NCI as a primary outcome measure, instead of a specific HAND. This approach was adopted because of the clinical utility of the dichotomous character of NCI, and also due to the controversy surrounding the distinction between asymptomatic NCI (ANI) and mild neurocognitive disorder (MND). Nevertheless, the HAND classification would have made it possible to integrate valuable information about the severity of the impairment.

Despite these limitations, and considering that our sample was from a single geographic area, the current work presents significant strengths. First, the models provided visual, easy-to-read outcomes that can be used immediately in clinical practice. Second, specific numeric cut-off points are offered for quantitative variables, also a major advantage in daily clinical practice. And third, different models are provided according to the level of accuracy and simplicity. Hence, clinicians and care providers are able to decide which models fit best in a specific setting. In fact, the degree of accuracy of the classifications was high. If we compare this feature with the accuracy provided by other screening tools for HIV-related NCI, it is clearly significant. Therefore, although the methodology presented should be seen as a novel approach when screening for HAND, the results generated can substantially help in the management and monitoring of neurocognitive complications in HIV-infected persons, particularly in those scenarios where clinical resources are limited. We fully recommend the application of neuropsychological test batteries for the rigorous assessment of neurocognitive functioning in HIV-infected individuals, but now we offer a potential screening method to identify the existence of risk and protective factors for NCI, considered as a supplementary resource for the therapeutic management of HAND.
